# Positive effects of green practices on the consumers' satisfaction, loyalty, word-of-mouth, and willingness to pay

**DOI:** 10.1016/j.heliyon.2023.e20353

**Published:** 2023-09-25

**Authors:** Darinka González-Viralta, Iván Veas-González, Francisco Egaña-Bruna, Cristian Vidal-Silva, Cristian Delgado-Bello, Claudia Pezoa-Fuentes

**Affiliations:** a Department of Administration, Universidad Católica del Norte, Angamos 0610, Antofagasta, 1240000, Antofagasta, Chile; b School of Videogame Development and Virtual Reality Engineering, Faculty of Engineering, University of Talca, Av. Lircay S/N, Talca, 3460000, Maule, Chile; c Escuela de Ingeniería Comercial, Facultad de Economía y Negocios, Universidad Santo Tomás, Antofagasta, 1240000, Antofagasta, Chile; d Instituto de Administración, Facultad de Ciencias Económicas y Administrativas, Universidad Austral, Valdivia, 5110566, Los Ríos, Chile

**Keywords:** Green practice, Satisfaction, Loyalty, WOM, Willingness to pay

## Abstract

Concern about green practices by companies and people has grown exponentially worldwide, considering them as a key element sustaining the development of competitive advantages. Given the present competitive, dynamic, and turbulent supermarket environment, managing environmental practices is essential for their survival. This study has two objectives: First, studying the relationship between green practices and customer satisfaction and, second, analyzing the impact of green practices and satisfaction on loyalty, word-of-mouth, and willingness to pay more via a quantitative study on a convenience sample of 458 customers from different Chilean supermarkets. Partial least square regression was used to analyze data. Results show the importance of green practices for developing satisfaction and different customer behaviors such as loyalty, word-of-mouth, and willingness to pay more. In addition, results prove satisfaction's positive impact on loyalty, word-of-mouth, and willingness to pay more. Those results also provide empirical evidence about the effects of green practices on the supermarket industry and, in this way, their advancement toward more sustainable management.

## 1 Introduction

Currently, the environment is considered a fundamental variable for the correct economic growth, the care of cities as countries, from ecosystems locally and globally [1], [2]. Companies are progressively emphasizing the importance of environmental sustainability, a movement that has amplified ecological consciousness and motivated individuals and groups to collaborate in shaping a more sustainable and ecologically conscious future. This collaboration involves the adoption of eco-friendly practices [3]. The Sustainable Development Goals (SDGs), defined by the World Organization of the United Nations (UN), where multidisciplinary challenges are posed, specifically number 14, refer to sustainable production and consumption towards the year 2030. Generating additional interest in investigating issues related to sustainability [4] from the point of view of individuals and firms [5], in this sense, sustainable practices (hereinafter green practices) are an essential element to achieve organizational sustainability [6].

The growing number of supermarkets in the last decade has increased energy consumption, mainly due to cooling systems [7]. In this context, chains such as Walmart and Carrefour are engaged in green practices via green supply chain management [8], sustainability, and the ecological entrepreneurial process by reducing wastes, particularly packing the products they trade [9] and fostering innovation, thus increasing energy efficiency to become more ecological supermarkets. Despite the importance of supermarkets and retailers, academic research pays very little attention to retail sustainability when implementing environmentally sustainable green practices [10], [11]. Hence, it is essential to examine in-depth green practices in this economic sector and, particularly, understand green practice mechanisms for establishing links with customers and supermarkets, considering variables such as satisfaction, loyalty, and word-of-mouth (WOM) [12], among others.

Green practices are methods businesses use to lessen or stop adverse environmental effects [13]. Research on green practices is varied and focuses mainly on tourism and hospitality sectors [14], [15], [16], [17]. Concerning retail, there are few studies on the use and development of green practices [18], [19], [20]. Therefore, this is an exciting gap to examine in greater depth. Retail sustainability results mainly consider aspects such as food origin and source [21]; texture, appearance, and taste [22]; and energy use and savings for production [23], [24], [25].

Research recently shows the importance of studying the positive effects of green practices on consumers. With this change generation, awareness of the environment is relevant, and satisfaction, loyalty, WOW, and willingness to pay are fundamental to understanding this new form of management. That allows us to deepen in contributing to issues related to green practices and customer satisfaction, satisfaction in loyalty, and new forms of marketing such as WOW and whether consumers are willing to pay more to have products that do not affect natural ecosystems.

The objectives that this research intends to achieve are divided into two: First, studying the relationship between green practices and customer satisfaction and, second, analyzing the impact of green practices and satisfaction on loyalty, WOM, and willingness to pay more in a supermarket context, via a quantitative study on a convenience sample of 458 Chilean customers, using multivariate analysis (SEM-PLS). Empirical data collection was made in June-December 2020. The main contribution of this study is the establishment of empirical evidence about the effects of green practices on the supermarket industry, using variables such as satisfaction, WOM, loyalty, and customer willingness to pay.

This paper is organized as follows: Section 2 deals with a systematic literature review focusing on green practices, satisfaction, loyalty, WOM, and willingness to pay. Section 3 refers to applied research methods. Section 4 presents the main results and their discussion. Section 5 presents related research work confirming the results of this work. Section 6 outlines this research's main conclusions and contributions. Finally, Section 7 describes this research's main implications and limitations.

## 2 Literature review and research hypotheses

### 2.1 Green practices

The term ‘green practices’ refers to “actions that reduce the environmental impact, such as eco-purchase and recycling” [26]. In this study, we will use the definition of [27]; green practices go far beyond environmental protection, emphasizing waste decrease to minimize their environmental impact. Green practices are positive for companies because they give rise to competitive advantages, placing them over the industry, favoring long-term relationships with customers [28], and improving financial performance by increasing customer loyalty, employees morale, retention rates, satisfaction, and stronger relationships with governments, thus leading to a better performance [29]. These practices allow companies to decrease long-term operational costs and improve brand image and reputation [30].

For a long time, consumers have been worried about environmental and health issues, particularly customers' and companies' green practice generation and use [31]. Schubert et al. [32] pose the importance of communicating and visualizing green practices, ecological products, and bags, and recyclable packages in the community, increasingly more familiar for consumers due to greater environmental awareness [33].

### 2.2 Satisfaction

Solimun and Fernandes [34] define satisfaction as the pleasure customers perceive when assessing a product or service. Studies in different contexts show a positive influence between green practices and satisfaction [16], [25], [35], [36]. That means that as green practices are generated, they will directly impact consumer satisfaction.

In the hospitality context, [36] confirm that green practices such as the use of installations consuming little electricity and soap-saving dispensers cause an impact on users' general satisfaction, and [16] point out that green practices cause a positive impact on guests' satisfaction. Implementing entrepreneurial green practices in retail influences customer satisfaction [18], [19]. The above leads to the following hypothesis:

**Hypothesis 1 (H1).** The green practices of supermarkets directly and positively affect consumers' satisfaction.

### 2.3 Loyalty

Loyalty is defined as “repeat purchasing frequency or the relative volume of same-brand purchasing” influencing cognition, affection, and behavior [37]. Luarn and Lin [38] indicate that loyalty results from peoples' positive consumption experience with an organization, creating favorable consumer attitudes. As a result, consumers gain confidence in a brand and perceive less risk for future purchasing [39]. According to [40], organizations that prioritize consumers and know their needs can develop a group of loyal consumers. Consumer loyalty is considered a strategic element because it is a source of income and organizational profitability in the long run, [41].

Incorporating environmental concepts in the marketing strategies of companies and organizations is considered a lever for improving loyalty [42]. According to the above several studies show the relationship between green practices and consumer loyalty [1], [16], [36], [43], [44], [45], [46], [47], [48], [49], [50], [51].

The work of [52] poses that supermarkets' green attributes, such as ecological products, brands, and prices have a positive relationship with customer loyalty. The above leads to the following hypothesis:

**Hypothesis 2 (H2).** Green practices in supermarkets have a direct and positive effect on consumer loyalty.

Choi and Hyun [53] point out that customer satisfaction directly and positively affects loyalty. The work of Torabi, Hamidi, and Safaie [54] indicates that “Customer satisfaction has a positive and direct effect on customer loyalty”. An example of this effect is a cafe chain, where customer satisfaction influences customer loyalty directly [43]. Other studies confirm customer satisfaction's direct and positive effect on customer loyalty, e.g., [1], [55], [56], [57]. This leads to the following hypothesis:

**Hypothesis 3 (H3).** Customer satisfaction has a direct and positive effect on supermarket customer loyalty.

### 2.4 Word-of-mouth

Word-of-mouth (WOM) is a method characterized by two-directional communication among customers, friends, or outsiders who provide useful information about a product, service, or specific brand, its acceptance being a powerful determinant for customers to acknowledge others' opinions [58]. The work of [59] defines WOM as “voluntary communications after consumer purchasing”. It occurs when consumers have strong feelings about an experience with a supplier, thus feeling motivated to share their experience with others [60].

Chang and Chang [61] report a positive relationship between green practices and WOM. They identified a significant WOM impact on consumers' decision to buy organic food. WOM effects, i.e., the ecological experience offered and customers' ecological experience, are significantly associated with consumers' behavior to purchase organic food. Lee, Han, and Willson [62] point out that environmental ventures conducted by hospitality enterprises probably encourage customers to engage in a positive WOM.

Recent studies such as [63] propose the following hypotheses for the relationship between green practices and WOM, “Willingness to pay significantly influences organic food decision-making” and “WOM significantly moderates the relationship between willingness to purchase and organic food decision-making”. Moise et al. [16] propose and confirm their hypothesis that “Green” practices adopted by hotels positively and significantly influence guests' positive WOM. The above leads to the following hypothesis:

**Hypothesis 4 (H4).** Green practices in supermarkets have a direct and positive effect on customer WOM.

The work of [64] states that customer satisfaction has a positive relationship with customers' WOM behavior. Also, online shopping websites influence customers' WOM satisfaction directly and positively [65]. Torabi, Hamidi, and Safaie [54] confirm that customer satisfaction has a direct and positive effect on customer WOM”. Then, the following hypothesis is posed:

**Hypothesis 5 (H5).** Satisfaction has a positive and direct effect on supermarket customer WOM.

### 2.5 Willingness to pay

Willingness to pay is the maximum amount of money a customer intends to pay for a particular product or service [66], [67]. According to [68], willingness to pay measures the value an individual assigns to purchase or experience of use in currency units. It is often considered one of the soundest results of loyalty and a vital measure of a brand value [69]. In addition, willingness to pay shows efficient brand management, representing a brand's ability to get a pay higher than its competitors [70].

Willingness to pay for green ventures has been studied in different contexts, including green products, e.g., [71], product packaging [72], [73], air travel, e.g., [74], [75], and hotels, e.g., [76], [77], [78]. Kang et al. [79] found that consumers concerned about the environment were willing to pay a premium for environmental practices at hotels. According to [80], consumers would be willing to pay more for products or services generating environmental strategies or practices, even when there is no direct benefit. Kuminoff, Parmeter, and Pope [81] claim that consumers may be willing to pay a premium for ecological products because they feel they are doing their share to help the environment. The above leads to the following hypothesis:

**Hypothesis 6 (H6).** Green practices in supermarkets have a direct and positive effect on consumer willingness to pay.

Homburg, Koschate, and Hoyer [68] found a positive relationship between satisfaction and willingness to pay. Several studies confirm the relationship between satisfaction and willingness to pay. For example, [82] found that willingness to pay is more strongly associated with tourist satisfaction. Lee, Graefe, and Hwang [83] found that the satisfaction of park visitors positively influences their willingness to pay. Satisfied customers who think that a specific company meets or exceeds their expectations would be willing to pay a premium for purchasing or experimenting with a brand [68], [84], [85]. The above leads to the following hypothesis:

**Hypothesis 7 (H7).** Customer satisfaction with a supermarket has a direct and positive effect on willingness to pay.

**Figure 1:**
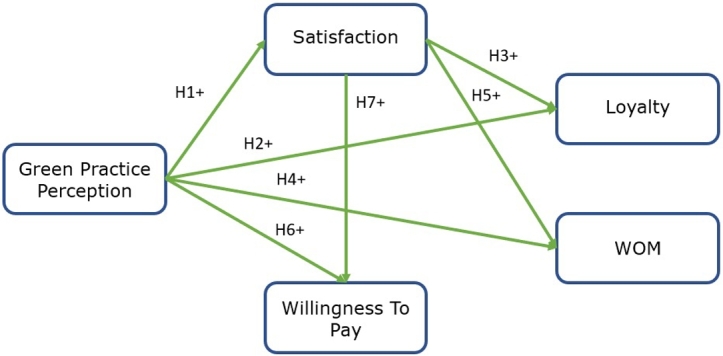
Research framework.

The model proposed here relates the constructs studied in Fig. 1.

## 3 Research methods

### 3.1 Methods, data collection, and sampling

This article applied a quantitative approach via a structured questionnaire to confirm the model hypotheses. The Ethical Scientific Committee from the Universidad Católica del Norte, Antofagasta, Chile, ethically approved the questionnaire before applying it (resolution 071/2020, protocol 024/2020). All the interviewed participants consented to participate in this study by answering the applied instrument.

Data were collected in Chile's second semester of 2020 using an online questionnaire administered to supermarket customers. This work used convenience sampling, and 458 valid questionnaires were collected.

Concerning participants' sociodemographic profile, Table 1 shows the sample distribution of classification variables: 27.1% men and 72.5% women. As to age range, most participants (38.6%) were 18-25 years old, followed by those 26-35 years old (32.8%), 36-45 years old (16.4%), 46-60 years old (10.7%), and finally, 60 years old or over (1.5%). Regarding education, 64.6% studied at college, 25.1% at secondary school, and 8.7% followed a degree program. Concerning geographical location, 40.8% lived in the north, 33.6% in the center, and 25.5% in the country's south. As to occupation, 41% were employed, 36.2% students, 11.4% householders, 10% unemployed, and 1.3% were retired. Regarding aggregated family income, 41.1% reported earning 3-5 times the country's legal minimum wage. Regarding the importance of green practices in the supermarkets studied, 72.1% reported that it is very important that the supermarkets they frequently visit generate or include green practices as part of their activities.

**Table 1:**
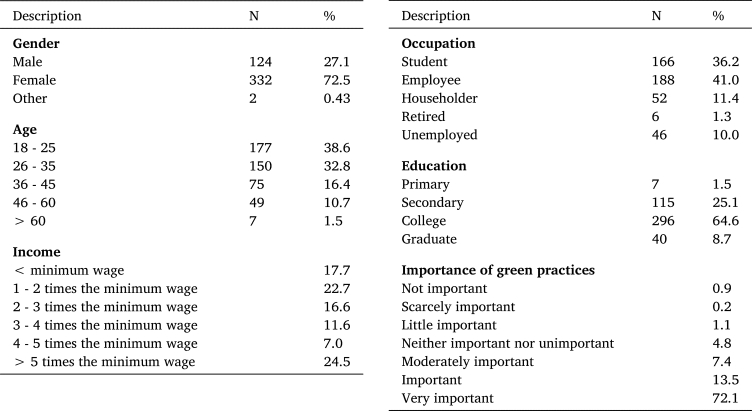
General sample.

### 3.2 Measurement

The items included in the variables were adapted to the context and scope of the study based on the different measurement scales used in previous studies. To measure the green practices of supermarkets, the one-dimensional 7-item scale of [86] was used. Concerning satisfaction, the 4 items proposed by [87] were used. To measure loyalty, 2 items of the scale proposed by [88] were used. The 2 items for measuring WOM were adapted from the scale proposed by [16]. Finally, 3 items for measuring willingness to pay were adapted from the scale proposed by [89]. All the variables were measured with Likert-type scales consisting of 7 options: 1 stands for “totally in disagreement”, while 7 represents “totally in agreement”. The items were previously translated from English to Spanish. Once the questionnaire was finished, a pilot trial was conducted on 50 subjects to ensure that respondents understood the meaning of each item. Based on the pilot trial results, some elements were slightly changed to make them more understandable. Table 5 in Appendix A presents the questionnaire's final version.

## 4 Results

We first performed Harmon's single-factor variance test for this study to examine common method bias. This result showed a common variation of 48%, less than the threshold value of 50% [90]. Therefore, it can be said that there are no common method bias concerns in the data. The data collected from this study were analyzed in two stages: the first to validate the measurement device and the second to evaluate the structural model [91]. The partial least squares method technique (PLS-SEM) and the SMARTPLS 3.2.7 program [92] were used. The PLS-SEM technique was used because it has been widely used by marketing researchers in recent years [93]. PLS-SEM is a technique to assess causal relationships between indicators or elements and latent constructs [94]. According to [92], PLS-SEM solves relevant problems that arise when proposing structural equations, such as impermissible solutions, factor indeterminacy, and the need for data normality. The reliability of the items, the internal consistency of the scale, and the convergent and discriminant validity were analyzed to test the reliability and validity of the instrument [95], [96]. Indicator reliability was assessed via loadings to accept an indicator as part of a specific construct with a loading higher than 0.70 [97]. Table 2 shows that all indicators meet this criterion. As to internal consistency, Cronbach's alpha and composite reliability were assessed, both criteria being over the 0.70 recommended value. Convergent validity was assessed with average variance extracted (AVE), all constructs of the structural model showing values over 0.50. Thus, each factor explains less than 50% of the assigned indicator variance [95]. Table 2 shows the reliability and convergent validity of the scales used for measuring the model variables.

**Table 2:** Confirmatory factory analysis.

Item	Standardized Loadings	Cronbach's Alpha	rho_A	Composite Reliability	Average Variance Extracted (AVE)
**Green Practice**		**0.932**	**0.937**	**0.945**	**0.712**
Green Practice 1	0.877				
Green Practice 2	0.858				
Green Practice 3	0.883				
Green Practice 4	0.912				
Green Practice 5	0.811				
Green Practice 6	0.715				
Green Practice 7	0.834				

**Loyalty**		**0.949**	**0.949**	**0.975**	**0.952**
Loyalty 1	0.976				
Loyalty 2	0.976				

**Satisfaction**		**0.968**	**0.968**	**0.977**	**0.913**
Satisfaction 1	0.922				
Satisfaction 2	0.968				
Satisfaction 3	0.972				
Satisfaction 4	0.959				

**Willingness To Pay**		**0.929**	**0.931**	**0.955**	**0.876**
Willingness To Pay 1	0.925				
Willingness To Pay 2	0.931				
Willingness To Pay 3	0.952				

**WOM**		**0.954**	**0.955**	**0.977**	**0.956**
WOM 1	0.978				
WOM 2	0.977				

### 4.1 Model assessment and reliability

To determine discriminant validity, criteria proposed by [98] and HTMT relationship method [99], [100] were used. According to [98], the square root of each variable AVE must be greater than the correlations with the other variables. Table 3 shows that all AVE square roots, represented in the main diagonal of the table, are greater than the correlation with every model construct. Concerning the HTMT relationship, [100] poses that the maximum value should be 0.90. Table 3 shows that all HTMT relationship values are below 0.90.

**Table 3:** Measurement model. Discriminant validity.

	Green Practice	Loyalty	Satisfaction	Willingnes To Pay	WOM
Green Practice	0.844	0.656	0.607	0.378	0.584
Loyalty	0.695	0.976	0.691	0.365	0.635
Satisfaction	0.636	0.721	0.956	0.463	0.796
Willingness To Pay	0.407	0.388	0.488	0.936	0.405
WOM	0.618	0.667	0.828	0.430	0.978

### 4.2 Structural model and hypotheses testing

The structural model was assessed globally and locally after assessing the measurement device and confirming its reliability and validity. For global assessment, the SRMR fit criterion indicating a good model fit with values lower than 0.08 was used [97]. The model proposed has a 0.039 estimated value (Table 4), indicating that expected frequencies are similar to observed frequencies, showing a good fit. For the local analysis of the structural model proposed, SmartPLS with a bootstrapping of 5000 subsamples was used [92]. R2 values obtained with bootstrapping indicate the model's ability to explain variable variability. According to [101], the higher the R2 values, the greater their predictive ability, the minimum value being 0.10. Table 4 shows that the R2 value of the dependent variables is over 0.10. The structural model's predictive relevance was also assessed using the Stone-Geisser Q2 test [102]. In this respect, [95] poses that the predictive relevance of the variables must be positive and show values greater than zero. Table 4 shows that all the values meet the parameters established and that the hypotheses previously formulated have an appropriate predictive value to assess the significance of the relationships above.

**Table 4:** Structural equation model results.

Hypothesis	Relationships	Standardized Beta	t-Value	p-value	Hypothesis
H1	Green Practice -> Satisfaction	0.607	17.193	0.000	Accepted
H2	Green Practice -> Loyalty	0.656	20.034	0.000	Accepted
H3	Satisfaction -> Loyalty	0.463	10.821	0.000	Accepted
H4	Green Practice -> WOM	0.584	16.692	0.000	Accepted
H5	Satisfaction -> WOM	0.700	16.68	0.000	Accepted
H6	Green Practice -> Willingness To Pay	0.378	8.272	0.013	Accepted
H7	Satisfaction -> Willingness To Pay	0.369	7.165	0.000	Accepted

R2 (LOY) = 0.566; R2 (SAT) = 0.368; R2 (WTP) = 0.230; R2 (WOM) = 0.650 Q2 (LOY) = 0.534; Q2 (SAT) = 0.332; Q2(WTP) = 0.198; Q2 (WOM) = 0.615 SRMR = 0.039.

**Figure 2:**
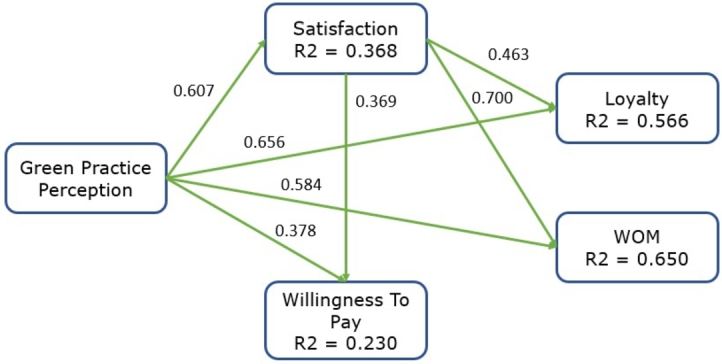
Results of the hypothesis model.

Results obtained with PLS-SEM allow accepting all the relationships proposed by the causal model. Regarding the first hypothesis, green practices positively and significantly affect satisfaction (B1 = 0.607; p < 0.001). Regarding the second hypothesis, green practices have a positive and significant effect on loyalty (B2 = 0.656; p < 0.001). The third hypothesis confirms consumer satisfaction's positive and significant effect on consumer loyalty (B3 = 0.463; p < 0.001). The fourth hypothesis confirms green practices' positive and significant effect on consumers' WOM (B4 = 0.584; p < 0.001). The fifth hypothesis confirms consumer satisfaction's positive and significant effect on customer WOM (B5 = 0.700; p < 0.001). The sixth hypothesis confirms the positive and significant effect of green practices on willingness to pay (B6 = 0.378; p < 0.05). Finally, consumer satisfaction's significant and positive effect on willingness to pay (B7 = 0.369; p < 0.001) is confirmed, see Fig. 2.

## 5 Discussion

This research has studied the role that green practices can play in determining aspects of organizational success from a marketing perspective in a context scarcely addressed in the literature - the supermarket industry. Thus, in the model, green practices have been defined as an independent and explanatory variable of the dependent variables: satisfaction, loyalty, WOM, and willingness to pay. Satisfaction has also taken the role of explanatory variable to evaluate its effects, together with green practices, on willingness to pay, WOM, and loyalty. Although the empirical literature supports a favorable effect of green practices on the marketing indicators mentioned above, most of this literature has been based on the context of the tourism industry and, more precisely, on the hotel industry [16], [25], [35], [36].

Concerning the effects on satisfaction (H1), the results in this research confirm that in the supermarket industry, green practices have a positive and significant effect on consumer satisfaction, reinforcing the results obtained in the literature. To some extent, this is because, in general terms, the supermarket industry in Chile is not characterized by a wide execution of green practices, thus transforming these practices into a significant differentiator for consumers. Berezan et al. [36] confirmed that green practices in the hotel industry favorably impact guest satisfaction. Likewise, [16] found that green practices significantly increase guest satisfaction. Regarding loyalty (H2), it is verified that green practices in supermarkets have a positive and significant effect on consumer loyalty, observing similar results to those proposed by [53] and [43]. To some extent, green practices contribute to building a trusting relationship between consumers and supermarkets, which may ultimately result in a more loyal consumer. Likewise, as in the research of [16], [61], [62], [63], this paper confirms that green practices positively and significantly affect WOM (H4). As [59] pointed out, this process occurs when the experience with the service generates strong feelings in consumers. In this work, we find evidence that when consumers' perception of green practices increases, their experience can lead to stronger and more permanent feelings, eventually transforming into positive comments about the supermarket and encouraging others to shop there. A consequence of all the above, and probably one of the most important effects, is the positive and significant effect of green practices on consumers' willingness to pay (H6). This demonstrates that green practices in supermarkets represent a value differentiator for customers. Similarly, [65] for 781 consumers in China found that, although most consumers have insufficient knowledge about green packaging, they have a strong willingness to pay more for this type of packaging.

On the other hand, concerning the role of satisfaction, the results in this work confirm a positive and significant relationship between satisfaction and consumer loyalty in supermarkets (H3), consistent with the findings of [53] and [43]. When supermarket service equals or exceeds consumer expectations, it will be conducive to greater consumer loyalty [103], in addition to the effect that green practices alone possess on loyalty. Also, the positive and significant relationship between consumer satisfaction and WOM (H5) is confirmed, which are similar results to those obtained by [64] and [54]; this means that to the extent that customers are satisfied with the supermarket, they will let their family and friends know, inviting them to shop there. Finally, the results confirm the positive and significant relationship between customer satisfaction and willingness to pay (H7), reinforcing what was pointed out by [68], [83], and [84]. Like green practices, satisfaction is also essential in explaining supermarket customers' willingness to pay. In this case, if the service experience in the supermarket significantly exceeds consumers' expectations and includes green practices, the perceived value of the service will increase, and in turn, the willingness to pay for it.

## 6 Conclusions

This research aims to evaluate the role of green practices in customer satisfaction and in turn, the role of these in loyalty, WOM, and intention to pay more. Green practices are an increasingly prevalent concept in the literature, largely due to the promotion of sustainable production and consumption by the UN (United Nations) through the Sustainable Development Goals. Although green practices have become increasingly important in the decisions of public and private organizations, the prevalence and effects of these practices may vary between productive activities and, in turn, between countries. Hence, this paper seeks to contribute to the literature from an industrial and territorial context that has been scarcely addressed, specifically the supermarket industry in Chile, a Latin American country. The supermarket industry represents an interesting case because of its wide coverage, reaching different market segments, and because it is in the incipient stage of developing green practices. Likewise, in the Chilean economy, the retail industry, particularly supermarkets, represents the country's main product distribution channel.

Although the level of development of green practices has been heterogeneous for industries/countries, an industry characterized by its comprehensive coverage and direct interaction with end consumers cannot ignore what is now a global trend, reaching more and more adherents in each country. This is confirmed by the statistically significant results of the model, where the hypotheses are verified, confirming that green practices play an essential direct role in satisfaction, loyalty, WOM, and willingness to pay. Likewise, satisfaction is essential in loyalty, WOM, and willingness to pay. In particular - and it stands out concerning the literature - the high predictive relevance of WOM. That is green practices and customer satisfaction explain to a large extent the variations of WOM in supermarkets, which is even more relevant in mass industries, where there are greater possibilities of interaction among customers. Finally, an interesting consequence of the results obtained is that consumers value implementing green practices by supermarkets, indicating that they would be willing to pay more when they perceive these practices. These results - from the consumers' valuation - indicate that green practices can contribute to sustainable organizational results.

## 7 Implications

### 7.1 Theoretical implications

The current study has provided a significant theoretical contribution; the findings have a novelty and a new contextual meaning. On the one hand, the concepts of green practices and the effects that these could have on these indicators have been introduced into the framework of relevant marketing performance indicators. Shows a close relationship between green practices and satisfaction, loyalty, WOM, and willingness to pay. Moreover, the theoretical and conceptual framework is applied in a context scarcely addressed in the literature on environmental practices and organizational effects. In this context, the supermarket industry, also called retail, demonstrates the critical role of green practices in performance outcomes from a marketing perspective. Practical implications Indeed, the model validated in this study strongly recommends that industry professionals and strategic decision makers of supermarket chain companies, as well as any competitor or substitute company, implement the development of new green practices, which, in addition to providing improvements in their corporate image and brand perception, will generate satisfaction, loyalty, a favorable WOM and a greater willingness to pay, in consumers. This may result in the long term, in a favorable financial effect within the logistics chain, as well as in competitive advantages, by promoting green practices in the organization. Although different green practices are recommended in this work, the actions of supermarkets that demonstrate concern, respect, and intention to protect the environment stand out. In addition, it is important to add that supermarket management teams should implement a proactive approach to monitoring environmental practices since green practices are constantly evolving due to the rapid technological change that has characterized the sector in recent times.

### 7.2 Policy implications

In terms of policies, the results of this work suggest that different green practices should be encouraged in retail companies due to the favorable effects that these practices can have on the performance of the organizations, which can ultimately lead to more significant economic growth of the sector and the country. In this sense, the promotion of green practices should not be an exclusive task of environmental institutions. It should also involve the institutionality of growth and productive development, due to the favorable effects that may arise in economic development. Additionally, the massive nature of the supermarket industry, its preponderance in the distribution channels, and its wide network of suppliers can promote a broad and rapid multiplier effect of green practices in companies of different categories, as well as a rapid and greater assimilation of the value of these practices by the population.

### 7.3 Limitations and possible areas for future studies

Regarding limitations, it should be considered that the sampling may not be representative of the entire national supermarket industry since the sampling design is non-probabilistic. On the other hand, the data collection instruments implemented in this work are perceptual and not observational or experimental, which may introduce some margin of error in the estimates. Although these results may present some limitations, the consistency, statistical significance, and high predictive capacity point to reliable conclusions. For future research, some of these limitations can be corrected, for example, by implementing a probabilistic sampling to safeguard representativeness. On the other hand, in the model's design, it is possible to introduce moderating sociodemographic variables and/or mediators of the loadings between the variables, allowing a better representation of the relationships between them. Likewise, it is possible to extend the context of analysis to other South American countries with economic characteristics like those of Chile.

## Additional information

Supplementary content related to this article has been published online at https://drive.google.com/drive/folders/1Q0gzC9_qnjFNwvxUPxPS83RgtVbXbqDv?usp=sharing.

## CRediT authorship contribution statement

Cristian Vidal Silva; Darinka González-Viralta; Iván Veas; Francisco Egaña; Cristian Delgado; Claudia Pezoa: Conceived and designed the experiments; Performed the experiments; Analyzed and interpreted the data; Contributed reagents, materials, analysis tools or data; Wrote the paper.

## Declaration of Competing Interest

The authors declare that they have no known competing financial interests or personal relationships that could have appeared to influence the work reported in this paper.

## Figures and Tables

**Table 5:** Questionnaire.

Construct	Items
Green Practice	This supermarket tries to improve its environmental performance.
	This supermarket respects the environmental norms defined in the law when carrying out its activities.
	This supermarket prioritizes environmental principles over achieving superior economic performance.
	This supermarket is concerned to respect and protect natural environment.
	This supermarket actively sponsors or finances environmental events.
	This supermarket sources many products from rural and economically less-developed areas.
	This supermarket is concerned to improve general well-being of society.

Satisfaction	Go to this supermarket was exactly what I needed.
	I was satisfied with decision of go to this supermarket.
	It was a wise choice go to this supermarket.
	It was a good experience go to this supermarket.

Loyalty	I would like to come back to this supermarket in the near future because it is environmentally friendly.
	This supermarket would be my first choice over other supermarkets because it is environmentally friendly

Willingness to pay	I am willing to pay higher price in this supermarket than for others supermarket of product.
	Even if the other supermarkets are priced lower. I will still buy in this supermarket.
	Even thought this supermarket seems comparable to other supermarkets I am willing to pay more.

Word-of-mouth	I would recommend this supermarket to other people.
	I would tell other people positive things about this supermarket.

## Data Availability

Data will be made available on request.
